# The association between atopic dermatitis and rosacea: a comprehensive review from comorbidities to pathogenic mechanisms

**DOI:** 10.3389/fimmu.2026.1837198

**Published:** 2026-06-05

**Authors:** Yuewei Zeng, Yanyan Feng

**Affiliations:** 1 Department of Dermatology, Sichuan University Affiliated Chengdu Second People’s Hospital, Chengdu, China; 2 Chengdu Second People’s Hospital, Chengdu, China; 3 West China School of Medicine, Sichuan University, Chengdu, China

**Keywords:** atopic dermatitis, rosacea, comorbidity, microbiome, innate immunity, adaptive immunity, neurovascula

## Abstract

**Objective:**

Atopic dermatitis (AD) and rosacea have traditionally been regarded as two distinct inflammatory skin disorders with divergent pathophysiology. However, emerging evidence has progressively revealed unexpected convergences between these conditions in clinical manifestations, comorbidity profiles and pathogenic mechanisms—raising the question of whether the apparent mechanistic overlaps represent true pathophysiological convergence—sharing common upstream drivers—or merely parallel, non- specific innate immune responses triggered by entirely distinct microbial and genetic factors? This distinction carries profound implications for the design of shared versus disease-specific therapeutic strategies. this review aims not simply to enumerate similarities but to critically evaluate whether the observed convergences represent true mechanistic overlap, or parallel but independent responses.

**Methods:**

Literature search strategy: A comprehensive literature search was conducted in PubMed, Embase and Scopus, the Cochrane Library between April, 2016 and April, 2026. The search strategy combined controlled vocabulary and free-text terms, including “ rosacea”, “ atopic dermatitis”, “ mast cells”, “ comorbidity”, “ microbiome”, “ innate immunity”, “ adaptive immunity”, and “ neurovascular”. Reference lists of relevant articles were also manually screened to identify additional eligible studies.

**Inclusion and exclusion criteria:**

Studies were included if they: (1) focused on atopic dermatitis and/or rosacea; (2) investigated relevant immunological mechanisms, comorbidities, or pathophysiological pathways; and (3) provided sufficient data to support qualitative synthesis. Both original research articles and high- quality reviews or meta-analyses were considered. Studies were excluded if they: (1) were duplicate publications; (2) lacked sufficient methodological detail or extractable data; or (3) were not directly relevant to the objectives of this review.

**Study selection and data extraction:**

Study selection and data extraction were performed independently by two reviewers. Titles and abstracts were initially screened, followed by full-text assessment for eligibility. Any discrepancies were resolved through discussion, and when necessary, a third reviewer was consulted to reach consensus.

**Study selection process:**

A total of 5381records were identified through database searching, with an additional 83 records identified through manual reference screening. After removal of duplicates, 3185records remained for title and abstract screening, of which 2864 were excluded due to irrelevance. A total of 321 full-text articles were assessed for eligibility, and 216 were further excluded for the following reasons: lack of relevance (n =117), insufficient data (n =53), or low methodological quality/non-original articles (n = 46). Ultimately, 105 studies were included in the qualitative synthesis. The study selection process is summarized in a PRISMA-style flow diagram (**Graphical Abstract**, below). Given the narrative nature of this review, no formal risk-of-bias assessment was performed.

**Results:**

Critical appraisal of the evidence reveals that both diseases share an upstream innate immune activation platform’ encompassing TLR2/TLR4 signaling, NLRP3 inflammasome activation, mast cell degranulation and neurovascular dysregulation via the CGRP/SP/VEGF/TRP axis. However, they diverge at the level of adaptive immune polarization: AD is dominated by Th2/ILC2 skewing with IgE sensitization and deficient antimicrobial peptide responses, while rosacea is characterized by Th 1/Th17 involvement with autonomous LL-37 overproduction as its primary amplification loop. Intriguingly, dupilumab-induced rosacea-like dermatitis suggests that these polarization states may not merely differ but actively compete, raising questions about the nature and limits of mechanistic overlap between the two conditions. This duality may challenge simplistic models of mechanistic overlap and has direct implications for differential clinical management. Future research should employ multi-omics approaches and prospective comorbidity cohorts to clarify causal pathways and translate mechanistic insights into optimized therapeutic strategies.

## Graphical Abstract

**Figure:**
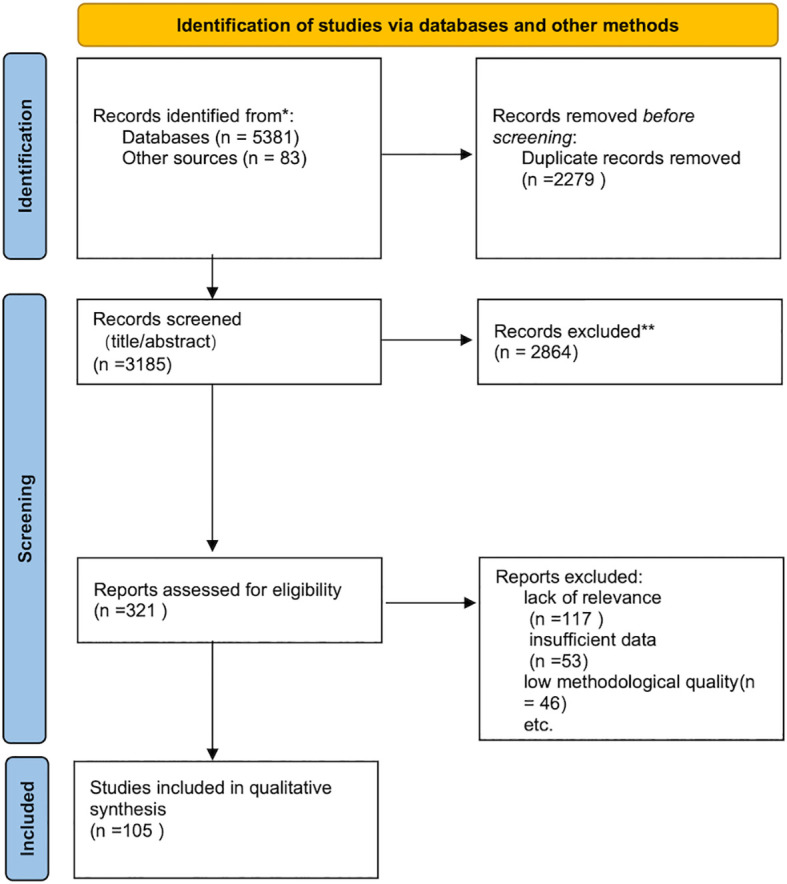
PRISMA flow diagram of study selection. A total of 5,381 records were identified through database searching and 83 additional records through manual reference screening. After removal of duplicates, 3,185 records remained for title and abstract screening, of which 2,864 were excluded as irrelevant. A total of 321 full-text articles were assessed for eligibility, and 216 were excluded due to lack of relevance (n = 117), insufficient data (n = 53), or low methodological quality or non-original articles (n = 46). Ultimately, 105 studies were included in the qualitative synthesis. No formal risk-of-bias assessment was performed due to the narrative nature of this review.

## 1 Introduction

**Figure 1:**
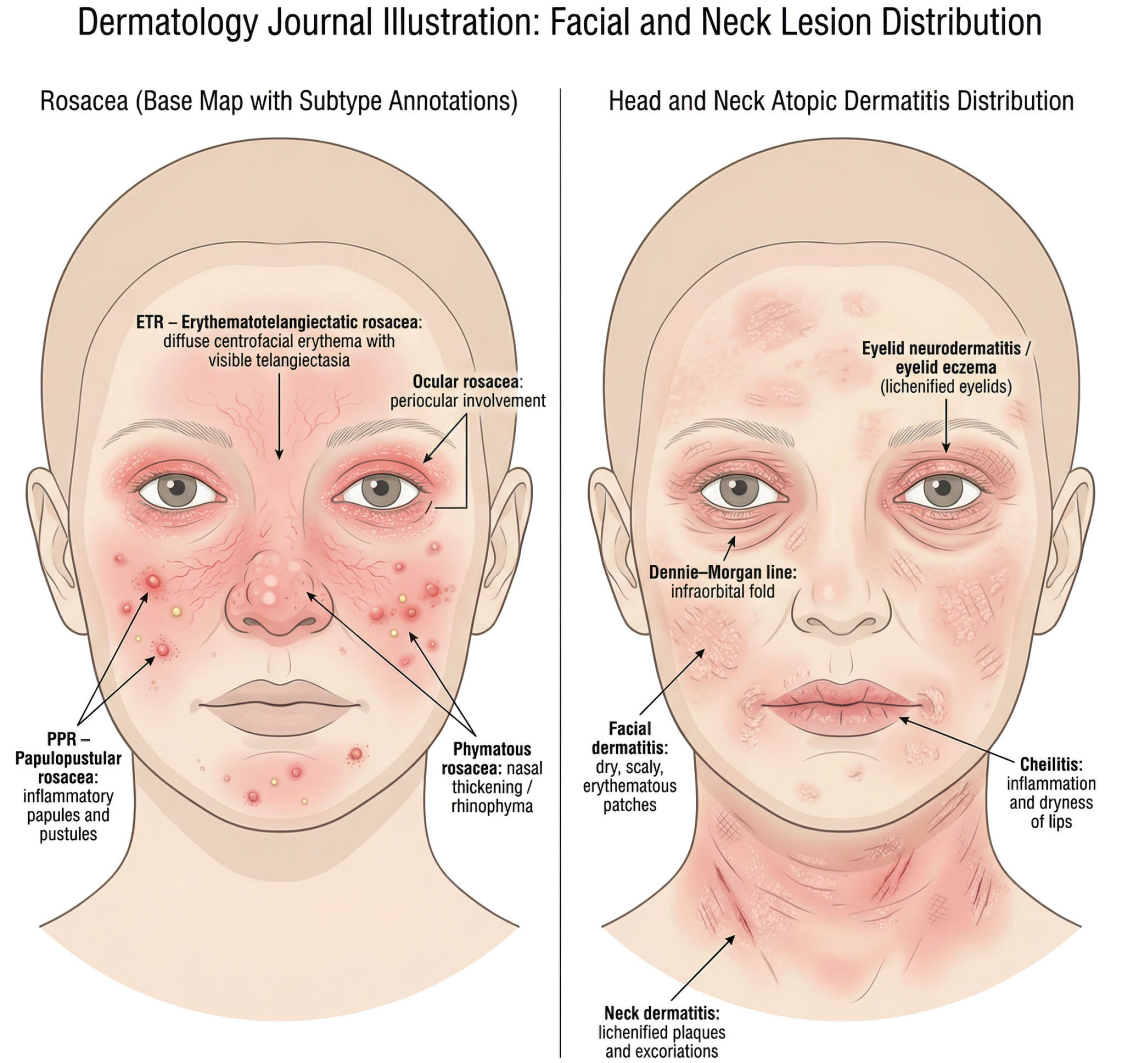
Facial lesion distribution in rosacea and atopic dermatitis. Left: Rosacea is characterized by persistent centrofacial erythema predominantly affecting the forehead, nose, and central cheeks, often accompanied by papules, pustules, and telangiectasia. Right: Head and neck atopic dermatitis presents with eyelid margin dermatitis, Dennie–Morgan folds, perioral dermatitis, and xerotic, scaly plaques with lichenification on the face and neck, frequently associated with pruritus and postinflammatory hyperpigmentation. Although both conditions may involve facial erythema and eyelid changes, rosacea is defined by central facial erythema with secondary papulopustular and vascular features, whereas atopic dermatitis is characterized by xerosis, lichenification, and typical periocular findings.

Atopic dermatitis (AD) and rosacea represent two prevalent chronic inflammatory skin diseases that impose substantial disease burden through high prevalence, persistent symptoms, cosmetic impairment, and recurrent flares, significantly affecting patients’ quality of life (1, 2). Within traditional dermatological frameworks, rosacea and atopic dermatitis have been conceptualized as entirely distinct and potentially mutually exclusive entities. AD is classically characterized by pruritic eczematous lesions driven by Th2-skewed immunity and *Staphylococcus aureus* colonization, whereas rosacea typically manifests as central facial erythema with inflammatory papules and pustules, associated with Th 1/Th 17 activation and Demodex proliferation (3, 4). However, emerging clinical and research evidence increasingly challenges this dichotomous view. Recent observations reveal substantial phenotypic and diagnostic overlap: rosacea and facial AD share overlapping distributions on the central face and exhibit similar symptom profiles (e.g., burning, stinging sensations) (5–7). (As shown in **Figure 1**) Notably, an increasing number of patients with facial dermatitis simultaneously meet the diagnostic criteria for both conditions. Meanwhile, the corresponding diagnostic labels may shift between rosacea and atopic dermatitis (AD) as the disease course progresses. This clinical convergence has prompted some experts to hypothesize that rosacea may represent a subtype within a broader facial AD-related inflammatory spectrum rather than an entirely independent disease. Supporting this framework, JAK inhibitors, which target broader inflammatory pathways beyond traditional Th2 mechanisms—have demonstrated efficacy in treatment-resistant rosacea in multiple case series (8, 9). Additionally, the emerging gut–skin axis model offers a potential unifying explanation: systemic immune priming from gut dysbiosis may lower individual immune thresholds, allowing local facial microenvironmental factors to determine which inflammatory phenotype becomes clinically dominant (10, 11). Meanwhile, paradoxical therapeutic responses introduce important caveats. Rosacea-like facial dermatitis has been increasingly reported in AD patients treated with dupilumab, an IL-4/IL-13 receptor α antagonist. Typical manifestations include central facial erythema, flushing, papulopustules, and burning sensations, even in individuals with no prior history of acne or rosacea, suggesting that Th2 pathway inhibition may facilitate Demodex proliferation and shift the immune milieu toward Th 1/Th 17 dominance (12, 13). This immune alteration may trigger IL-17–driven inflammation implicated in rosacea pathogenesis and lead to meibomian gland dysfunction in affected patients (14). This phenomenon suggests that AD and rosacea may involve competing immune polarization states within shared facial susceptibility, rather than representing a simple disease substitution or continuous spectrum. However, this interpretation remains speculative, and alternative explanations, including microbiome shifts or treatment-related local effects, cannot be excluded. Given these convergent yet seemingly contradictory observations, a comprehensive critical analysis is warranted. This review synthesizes evidence for shared and divergent pathogenic mechanisms between AD and rosacea, examines how therapeutic responses illuminate disease biology, and proposes an integrated framework to clarify their relationship and guide future therapeutic development.

## 2 Common comorbidities with focus on autoimmune diseases

AD shares 39 genetic loci with inflammatory bowel disease (IBD), and genomic studies have identified HLA haplotypes as common genetic susceptibility factors between AD and autoimmune diseases (15). Rosacea patients similarly exhibit enrichment of multiple HLA alleles associated with autoimmunity (16). Epidemiological data show that both conditions are enriched for autoimmune comorbidities(IBD, alopecia areata and Vitiligo) and psychiatric disorders (17–19), yet the association patterns are not identical: AD more strongly clusters with IBD and alopecia areata across ages, whereas rosacea shows clearer age/sex-stratified autoimmune associations (e.g., vitiligo after midlife, male-predominant alopecia areata) (20, 21). Neuropsychiatric comorbidity is also common in both (22, 23) but causality remains uncertain. Overall, the observed clustering of AD and rosacea with immune-mediated comorbidities suggests convergent epidemiological risk. Genetic and immunogenetic findings further support the plausibility of shared upstream susceptibility (e. g., HLA-associated predisposition and genetic overlap with IBD).

Nevertheless, comorbidity overlap does not by itself establish shared downstream pathogenic mechanisms. First, most studies rely on nationwide administrative registers or retrospective cohort designs, where diagnostic misclassification between facial AD and rosacea may inflate apparent comorbidity estimates (As shown in **Table 1**). In addition, treatment-related confounding is rarely addressed; patients receiving systemic immunosuppressive or immunomodulatory therapies (e.g. ciclosporin, dupilumab) may exhibit substantially altered comorbidity risk profiles compared to untreated individuals, yet stratification by treatment history is often lacking. Furthermore, the causal direction of gut–skin associations remains insufficiently defined for rosacea. While Mendelian randomization studies have begun to address this question in AD (24, 25), comparable causal inference analyses for rosacea–IBD relationships remain limited. Finally, psychiatric comorbidity in both conditions may be more plausibly explained by psychosocial burden—including chronic facial involvement, sleep disturbance, and social stigma—than by a shared neuroimmune mechanism, although current evidence cannot clearly distinguish between these explanations (26).

**Table 1:** Comparison of differential diagnosis and management of facial rosacea-like dermatitis.

*Diseases*	Differentiating clinical features	Recommended work-up	Management
*Rosacea (* 99 *)*	Rhinophyma Centro facial region; flushing	Dermoscopy Reflectance confocal microscopy Histopathologic examination Computer-aided diagnostic system	Antimicrobial topical preparations Topical eye medications Phototherapy and other physical therapies Operative therapy
*Head and neck atopic dermatitis (* 100 *)*	facial pigment changes Dennie–Morgan infraorbital folds. Hertoghe sign Subauricular and postauricular fissures Dirty neck (DN)	Fungal microscopy and fluorescence testing Malassezia-specific IgE Tape-strip biopsy for Demodex detection Wound swab for bacterial identification Patch testing Phototesting Photopatch testing	Emollients/moisturizers Physical cooling Topical corticosteroids (TCS) Topical calcineurin inhibitors (TCI) Phosphodiesterase-4 inhibitors (PDE4 inhibitors) Dupilumab JAK inhibitors antimicrobial therapy
*Dupilumab-associated rosacea-like dermatitis (* 101 *)*	Patient on dupilumab Unilateral Neck involvement (102)	Skin Scraping for Malassezia Yeast/Demodex Mites Serology Screening Biopsy Patch Testing	Allergen Avoidance Topical Corticosteroids (TCS)/Topical Calcineurin Inhibitors Antifungals; Antibiotics Discontinuing Dupilumab
*corticosteroid-induced rosacea-like dermatitis* *(CIRD) (* 103 *)*	Burning sensation, blushing, erythema, heat intolerance Prolonged or inappropriate TCS use Women > men	Clinical	Steroid-sparing therapy Hyperpigmentation depigmenting agents Anti-inflammatory therapy Antihistamines for pruritus Antibiotics for infections Physical therapy

Taken together, these limitations suggest that shared comorbidity patterns likely reflect convergent epidemiological risk profiles but should not be interpreted as definitive evidence of shared biological disease mechanisms, highlighting the need for further mechanistic and longitudinal studies.

## 3 Mechanistic convergence and divergence

**Figure 2:**
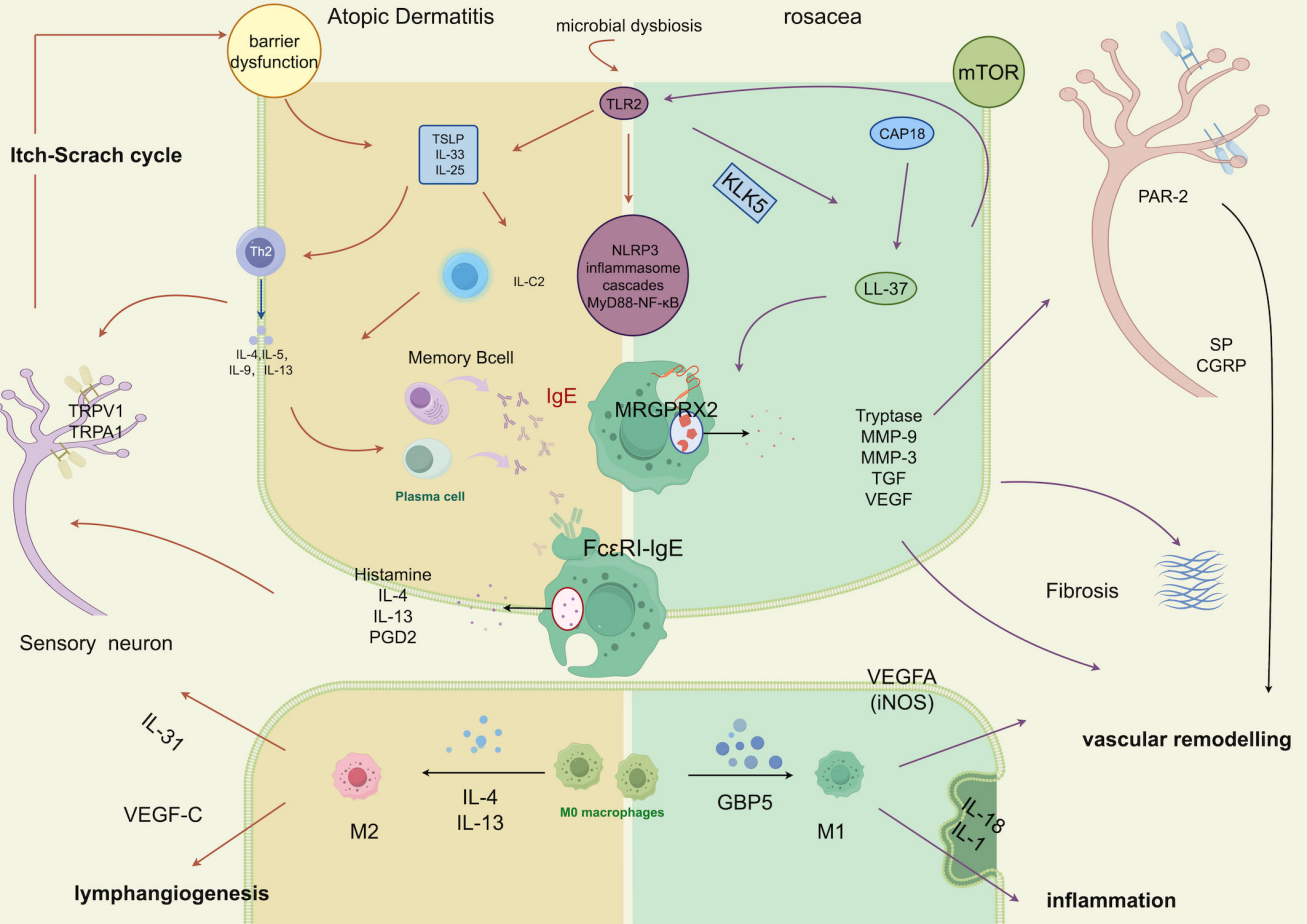
Three-layer model of shared pathogenesis in rosacea and atopic dermatitis. The model illustrates three interconnected levels. (1) Upstream triggers include microbial dysbiosis and skin barrier dysfunction, which are common to both diseases. (2) The amplification layer links triggers to disease outcomes and involves mast cells and macrophages, which exhibit context-dependent behavior. In atopic dermatitis, mast cells are primarily activated via IgE-mediated pathways and promote type 2 inflammation, whereas in rosacea they are activated through MRGPRX2 and are associated with innate immune dysregulation. Macrophage polarization also differs, with M1 predominance in rosacea and M2 polarization in atopic dermatitis. (3) These processes collectively drive downstream inflammatory outcomes.

A critical appraisal of the mechanistic literature reveals that AD and rosacea are neither pathophysiologically identical nor wholly independent. We propose that their relationship is best understood through a three-tier hierarchy (1): upstream triggers and innate sensing (e.g., microbiome dysbiosis, TLR/NLR activation) (2), intermediate amplification circuits (e.g., LL-37, mast cells, neurovascular signaling),and (3) downstream effector programs defined by adaptive immune polarization(As shown in **Figure 2**). Although substantial overlap exists at the first two levels, these commonalities may not be disease-specific and are shared across many chronic dermatoses. The key divergence occurs at the third level, where Th2 polarization in AD versus Th 1/Th 17 polarization in rosacea determines clinical phenotype and therapeutic response.

### 3.1 Shared upstream triggers: real but non-specific

#### 3.1.1 Skin microbiome dysbiosis

Both atopic dermatitis (AD) and rosacea exhibit microbial dysbiosis characterized by two convergent pathogenic features. First, significantly reduced microbial diversity disrupts commensal networks, accompanied by dominant species replacement. Key commensals (coagulase-negative staphylococci, Streptococcus spp., Corynebacterium spp., Propionibacterium spp., and Proteobacteria) decrease in relative abundance, while pro-inflammatory or opportunistic pathogens gain dominance (4, 27). *Staphylococcus aureus* colonizes 90% of AD lesions with 70-90% relative abundance, correlating with disease severity (28). In rosacea, Demodex mites and their commensal Bacillus oleronius molecular patterns enrich within the follicular-sebaceous unit niche (4). This microbial shift releases abundant microbe-associated molecular patterns (MAMPs)—lipoproteins, lipoteichoic acid, lipopolysaccharides, peptidoglycan fragments (29, 30), which subsequently activate TLR2/TLR4 on keratinocytes and Langerhans cells (35, 36, 105), thereby triggering MyD88-NF-κB and NLRP3 inflammasome cascades (31, 32). Mechanistically, AD involves *S. aureus*-derived phenol-soluble modulins (PSMs) promoting lipoprotein vesicle shedding, synergizing with barrier defects to potentiate TLR2 activation. In rosacea, Demodex-associated B. oleronius lipoproteins, lipoteichoic acid, and 62/83-kDa antigens activate TLR2/4 (31, 33) TLR2/TLR4 activation triggers the IRAK–TRAF6–TAK1 signaling axis, which subsequently activates NF-κB and MAPK pathways, leading to AP-1 family transcription factor activation and/or enhanced stability of associated mRNAs (34).

Disease-specific divergence already emerges: In AD, TLR activation induces keratinocytes to release ‘alarmins ‘ such as cytokines of the IL- 1 family, TSLP and IL-33, which prime a Th2 bias (35). while rosacea keratinocytes upregulate KLK5/7, processing hCAP18 into pro-inflammatory LL-37 and increasing the expression of β-defensins and VEGF (36). These processes couple with JAK/STAT pathways to suppress barrier genes and promote inflammatory amplification (37). Rosacea involves JAK2/STAT3 activation triggering pro-inflammatory cytokines (TNF-α, IL-6, IL-8), mast cell degranulation, and release of histamine/neuropeptides, amplifying vasodilation, neural sensitization, and chronic inflammation (38).

#### 3.1.2 Systemic gut-skin axis effects

The influence of microbe-host interactions extends beyond the skin’s localized effects. Recent Mendelian randomization studies have confirmed the causal role of the gut-skin axis in inflammatory skin diseases (39). The gut microbiota influences the skin through two parallel pathways: ① Microbial metabolites (including alterations in metabolic capacity and metabolite levels) modulate systemic immune responses, disrupting skin homeostasis (40, 41) ② the gut-brain-skin neuroendocrine signaling pathway: serial neuroendocrine signaling via the HPA axis, multiple neurotransmitters, neuroactive metabolites, and tryptophan metabolism (42). Probiotic interventions targeting the gut microbiome have improved rosacea symptoms (43), and lactobacillus-based formulations significantly reduce SCORAD scores in pediatric AD patients (44), further substantiating the pivotal role of gut microbiota in rosacea and AD pathogenesis. The microbiota-neuro-immune interaction forms an integrated framework for understanding both conditions. Abnormalities in this interaction loop are also the common molecular basis for itching in AD patients and flushing in rosacea patients.

### 3.2 Shared amplification modules

**Figure 3:**
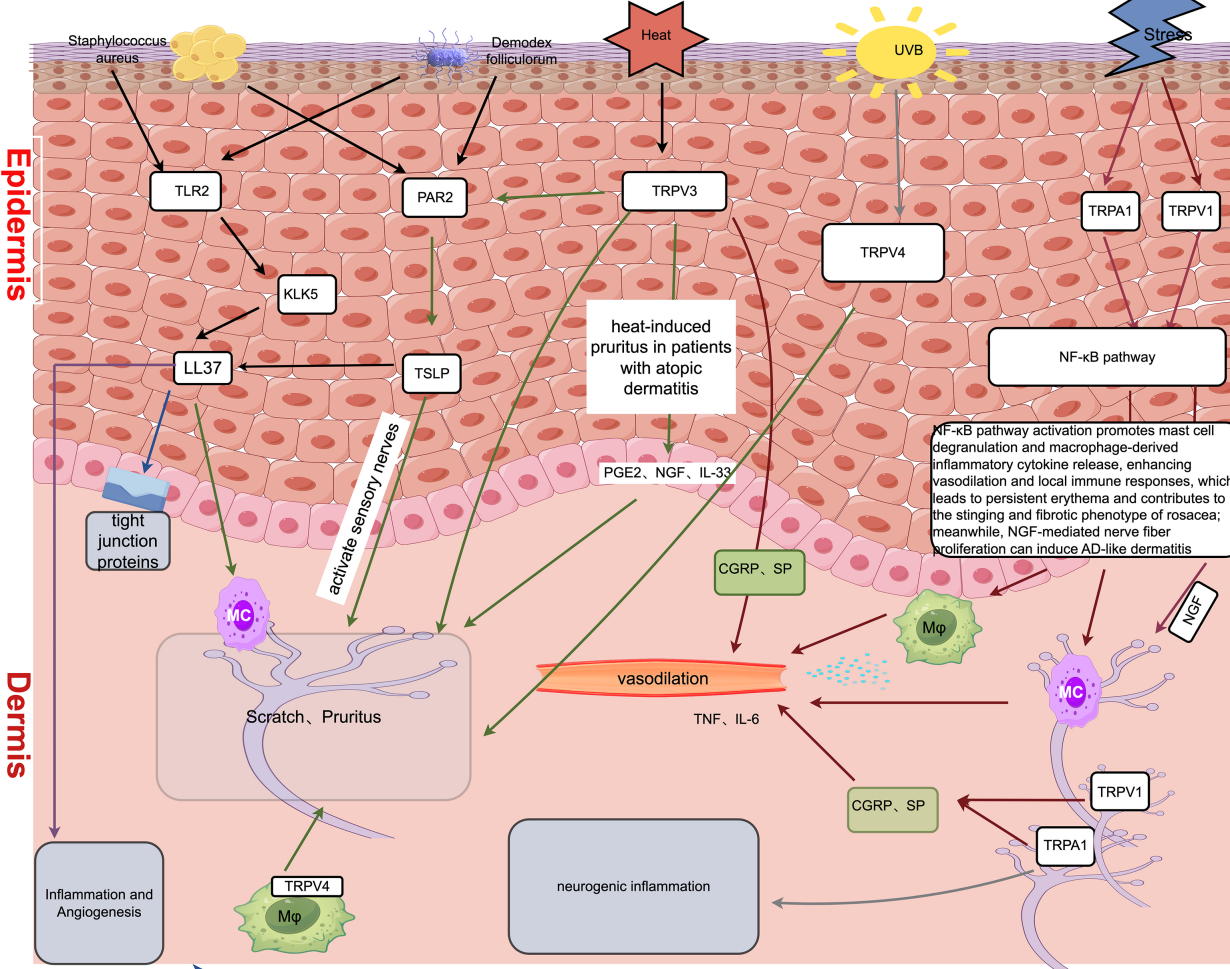
Role of mast cells in rosacea and atopic dermatitis. Microbial stimuli, UVB radiation, and allergens activate epidermal pathways including TLR2, NF-κB, and NLRP3, leading to the production of LL-37, neuropeptides, and IgE, which trigger mast cell activation and degranulation. Activated mast cells release mediators such as histamine, proteases, IL-31, CGRP, substance P, and VEGF, promoting sensory nerve activation (pruritus, burning, stinging) and vascular responses (vasodilation and angiogenesis), resulting in erythema, telangiectasia, papules, pustules, and rhinophyma. In parallel, mast cells recruit and modulate eosinophils, neutrophils, macrophages, and Th17 cells, amplifying and sustaining chronic inflammation. These processes highlight the central role of mast cells in neuro-immune-vascular interactions in both diseases.

**Figure 4:**
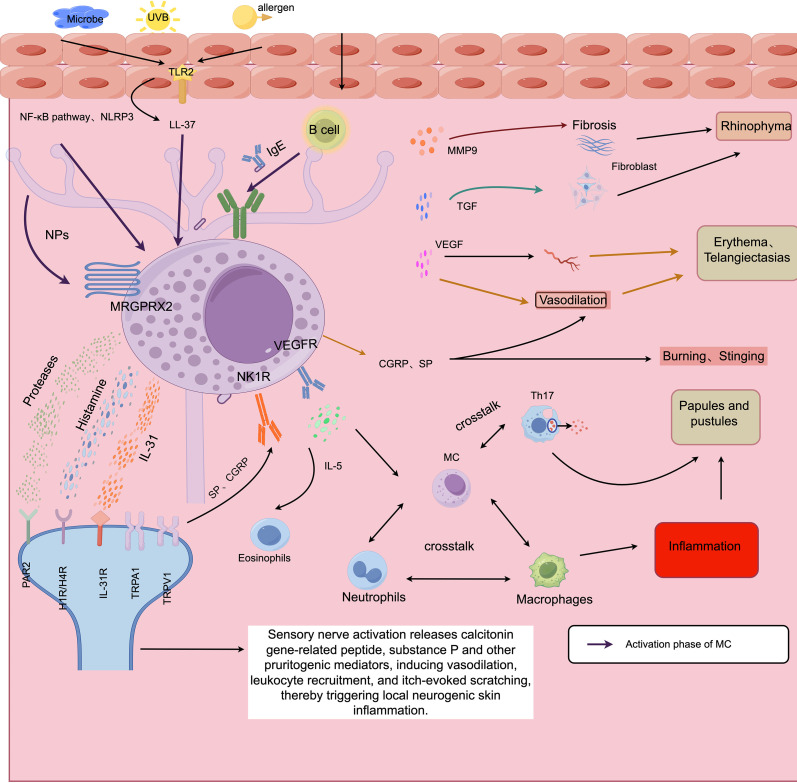
TRP channel–mediated neurogenic inflammation and pruritus in rosacea and atopic dermatitis. Environmental stimuli activate transient receptor potential (TRP) channels on keratinocytes and sensory nerve endings, initiating three interconnected cascades. (1) Neurogenic inflammation: TRP activation induces the release of neuropeptides (CGRP and substance P), leading to vasodilation and plasma extravasation. (2) Inflammatory signaling: TRP activation in keratinocytes triggers NF-κB–dependent pathways, promoting the production of inflammatory mediators (PGE_2_, NGF, IL-33, TNF, IL-6) and activation of mast cells and macrophages. Heat-induced pruritus in atopic dermatitis is mediated by the TRPV3–PAR2–TSLP axis. (3) Chronic outcomes: Sustained activation results in vasodilation (facial flushing in rosacea and erythema in both conditions), pruritus or pain, and tissue remodeling, including fibrosis in rosacea and NGF-mediated nerve fiber proliferation in atopic dermatitis.

These are cellular and molecular components that are activated in both AD and rosacea. However, their downstream effects depend on the local immunological context and are therefore not fully interchangeable between diseases. In this study, we focus on three major modules: macrophages, mast cells/LL-37 and the TRP–PAR-2–VEGF neurovascular axis. (As shown in **Figures 3**, **4**).

#### 3.2.1 Macrophages

Macrophages are pivotal innate immune effectors that contribute to atopic dermatitis and rosacea by modulating cytokine networks, vascular reactivity, and tissue remodeling. Depending on the local immune microenvironment, macrophages differentiate into distinct M1 and M2 phenotypes (45).

In rosacea, M1 polarization dominates. Macrophage infiltration is associated with disease severity, as reflected by Clinical Erythema Assessment (CEA) and Investigator Global Assessment (IGA) scores (46). Functionally, M1 macrophages promote vascular remodeling through STAT3/NF-κB-dependent upregulation of vascular endothelial growth factor A(VEGFA) and nitric oxide (NO)-mediated vasodilation driven by inducible nitric oxide synthase (iNOS) (47–49).

In contrast, AD is characterized by M2 polarization, largely driven by Th2 cytokines. M2 macrophages show increased expression of CCL13/CCL18 and further amplify type 2 inflammation through bidirectional crosstalk with T cells, dendritic cells, and fibroblasts (50). They also secrete IL-31, under cooperative regulation involving the TSLP–periostin–basophil network and Th2 cytokines (IL-4/IL-13),which activates neural pathways and contributes to pruritus (51). Additionally, in AD,IL-4-conditioned keratinocytes upregulate MCP-1 to recruit VEGF-C-positive macrophages that activate lymphangiogenic programmes (52).

#### 3.2.2 Mast cells and LL-37: a coupled amplification loop

Mast cells are robustly activated in both atopic dermatitis and rosacea (53, 54), yet the upstream signals and receptor pathways involved differ between them. Specifically, these processes are mediated by two distinct pathways: the IgE–FcϵRI–histamine axis and the MAS-related G protein–coupled receptor X2–tryptase pathway (55). It is this receptor-level divergence, rather than any difference in the mast cell itself, that determines the downstream effector profile.

##### 3.2.2.1 In atopic dermatitis: allergen-driven IgE–FcϵRI axis

In AD, mast cell activation is primarily IgE-mediated. The Th2/ILC2 polarization program drives sustained IgE class-switching in B cells (56); the resulting allergen-specific IgE constitutively occupies FcϵRI on mast cell surfaces. Re-exposure to allergen, triggering immediate degranulation with release of histamine, prostaglandin D2, and leukotrienes, followed by *de novo* synthesis of IL-4, IL-13, and IL-31,TSLP (57–59). The downstream consequences are distinctly pruritogenic and Th2-amplifying (55). Histamine acts on H1 and H4 receptors expressed on sensory neurons to lower itch thresholds and sensitize TRPV1 and TRPA1 channels, establishing a direct neuro-immune coupling that drives the characteristic pruritus of AD (55, 60) Meanwhile, Various cytokines, such as IL-4 and IL-13, released by mast cells further enhance the polarization of M2 macrophages and continue to stimulate IgE production, thereby creating a self-sustaining Th2 feedback loop. With regard to vascular regulation, in atopic dermatitis, mast cells primarily regulate VEGF production through prostaglandin E2, adenosine, and IL-9/IL-9R signaling, contributing to lymphangiogenesis and angiogenesis (61).

In this context, LL-37 acts as a peripheral modifier rather than an activation driver.LL-37 is paradoxically deficient in AD lesional skin, contributing to impaired antimicrobial defense rather than inflammatory amplification (62), Although LL-37 can activate mast cells to release interleukin (IL)-31, granulocyte-macrophage colony-stimulating factor (GM-CSF), and nerve growth factor (NGF) (63, 64), LL-37 may reduce itching in patients with AD by inducing the expression of semaphorin 3A (65). LL-37 therefore plays a secondary, context-dependent and partially self-limiting role in AD mast cell biology—a sharp contrast to its function in rosacea.

##### 3.2.2.2 In rosacea: LL-37-driven MRGPRX2–tryptase axis and the autonomous amplification loop

In rosacea, mast cell activation is largely IgE-independent and driven by LL-37, the overproduction of which is induced via a TLR2/KLK5 pathway (31). LL-37 then activates MRGPRX2, a non-IgE G protein-coupled receptor, expressed on mast cells (66, 67), triggering mast cells degranulation and tryptase release. The released tryptase then activates protease-activated receptor 2 (PAR-2) on sensory nerve terminals, inducing the release of calcitonin gene-related peptide (CGRP) and substance P (68, 69), These neuropeptides promote vasodilation, plasma extravasation, and leukocyte recruitment (70). Critically, this PAR-2–CGRP/substance P signaling feeds back onto mast cells via a MRGPRX2 and NK1R receptors, sustaining further MRGPRX2-mediated degranulation and establishing a bidirectional neuro-mast cell amplification loop (69), perpetuating mast cell degranulation independent of allergen or IgE-mediated signals. This MRGPRX2–tryptase–PAR-2 loop is self-sustaining and IgE-independent, This explains why rosacea persists even without allergen sensitization, responds poorly to antihistamines, and instead requires interventions that target LL-37 production or MRGPRX2 signaling.

In addition to the main activation loop mentioned above, LL-37 further amplifies the pathology of rosacea through three coupled mechanisms (1): NLRP3 axis: LL-37 triggers NLRP3 assembly and promotes caspase-1-mediated the maturation and release ofIL-1β and IL- 18 (71), thereby stimulating T-cell proliferation and Th 17 differentiation (72, 73) (2). In the vascular axis, LL-37 activates endothelial VEGF signaling through FPRL1/mTORC1 (74), upregulates JAK2/STAT3, thereby promoting mast cell VEGF production and resulting in sustained hemangiogenesis and telangiectasia (37) (3). In the autonomous amplification loop, on the one hand,LL-37 amplifies Th 17 inflammation by inducing Th 1/Th 17 polarization-associated genes (75), on the other hand, IL-17/IL-22 activates keratinocyte cathelicidin pathways via vitamin D3 signaling, promoting LL-37 production and creating a self-reinforcing LL-37-Th 17-LL-37circuit (36, 76). The net result is that in rosacea, LL-37 is not merely a shared amplifier but the primary disease driver—establishing and maintaining the Th 1/Th 17 microenvironment that distinguishes rosacea from AD.

##### 3.2.2.3 Therapeutic implication

Because mast cell receptor–level activation axes diverge between diseases, pathway-matched therapies do not necessarily translate across conditions. Anti-IgE therapy (omalizumab) can rationally inhibit the FcϵRI-driven mast cell activation contributing to atopic dermatitis (AD),yet it cannot suppress the LL-37/MRGPRX2–tryptase circuitry that sustains rosacea. Conversely, targeting the LL-37 pathway—e.g., TLR2 inhibitors, KLK5 inhibitors, and mTORC1 blockade (rapamycin, celastrol) (77)—may disrupt the dominant activation route in rosacea, but is unlikely to provide comparable benefit in AD. In AD, where LL-37 can exert paradoxical antipruritic effects via semaphorin 3A, inhibiting this axis could theoretically worsen itch.

#### 3.2.3 TRP channels and the PAR-2/CGRP/VEGF neurovascular axis

##### 3.2.3.1 Transient receptor potential channels

TRP channels (TRPV1, TRPA1, TRPV3, TRPV4) are non-selective Ca²^+^ channels abundantly expressed on cutaneous sensory nerve terminals and keratinocytes (78). Activated by thermal stimuli, reactive oxygen species(ROS), inflammatory mediators, and PAR-2 signaling, they triggers similar neurogenic inflammation and neurovascular responses in both diseases (79).

In both conditions, the activation of TRPV1/TRPA1 on sensory nerves increases the influx of Ca²^+^/Na^+^, which releases CGRP and SP. These substances induce vasodilation, enhanced permeability and plasma extravasation (80, 81). keratinocyte TRPV3 activation similarly mediates heat-induced vascular regulation through local CGRP release (82). TRP activation also triggers NF-κB-mediated COX-2, IL-1β, IL-8, TNF-α,PGE2, NGF, and MMP upregulation (60, 83).

Disease-specific divergence: In atopic dermatitis (AD), IL-13, IL-31, and TSLP can upregulate TRPV1 and TRPA1, driving chronic itch through sensory neuron pathways (60). In addition, keratinocyte TRPV3 activation may contribute to heat-induced itch in AD patients by promoting TSLP release, activating sensory nerves, and directly releasing pruritogenic mediators (84, 85). In rosacea, TRP activation is preferentially linked to mast cell degranulation and macrophage activation (86, 87), and is thus directed toward vasodilation and facial flushing. Notably, Some TRPV3 and TRPV4 channels may also be involved in the fibrotic process of rosacea (78, 88).

##### 3.2.3.2 VEGF

VEGF is implicated in both atopic dermatitis (AD) and rosacea as a key pro-angiogenic mediator. In atopic dermatitis (AD), VEGF levels are elevated in both the serum and lesional skin compared with healthy controls, and these increases correlate with disease severity (89).Similarly, VEGF expression in rosacea lesional areas is markedly higher than in non-lesional regions (90), supporting vascular involvement in both diseases. Genetic evidence further supports a shared contribution of VEGF signaling, including VEGF polymorphisms in rosacea (91) and the rs448012 single nucleotide polymorphism in FLT4 (VEGFR-3) gene in atopic dermatitis (92). Finally, interventions that reduce VEGF activity have been shown to improve disease-related skin inflammation and angiogenesis in both contexts (93, 94). Overall, these findings support VEGF as a shared angiogenic module contributing to vascular generation/remodeling in AD and rosacea.

### 3.3 Distinct immune microenvironments as the key determinants

The mechanistic evidence reviewed above highlights a key interpretative challenge: the same upstream molecular machinery-TLR2/NLRP3 activation, macrophage activation, mast cell degranulation,KLK5-mediated LL-37 processing,PAR-2/TRP channel signaling, and neuropeptide release-operates in both AD and rosacea, yet produces distinct clinical outcomes: persistent centrofacial erythema and telangiectasia in rosacea, versus heat/sweat-triggered pruritus and eczema in AD. We propose that this apparent paradox is best resolved by recognizing that the dominant immune microenvironment acts as a central regulator, redirecting shared upstream signals toward disease-specific amplification programs.

#### 3.3.1 Th2/ILC2 dominance in atopic dermatitis

In Atopic dermatitis, the dominant program is Th2/ILC2-mediated, and characterized by IL-4/IL-13 signaling, elevated TSLP, and IL-31 overproduction. In this context, dendritic/Langerhans cell presentation initiates OX40L-OX40 and JAK-STAT signaling, inducing Th2/Th22.

polarization (IL-4/IL-13, IL-22), while inhibiting keratinocyte differentiation and barrier proteins. This exacerbates TEWL (38), IL-4/IL-13 signaling also upregulates TRPV1 and TRPA1 on sensory nerves, driving C-fiber itch sensitization. Furthermore PAR-2 activation on keratinocytes releases TSLP and PGE2, which further amplify itch circuits rather than vascular remodeling (69, 84).

#### 3.3.2 Th 1/Th 17 involvement and autonomous LL-37 amplification in rosacea

In rosacea, transcriptomic studies confirm activation of Th 1/Th 17 polarization pathways. Consistent with this, immunohistochemistry of lesions demonstrates high expression of IFN-γ and IL-17A, with persistently elevated serum IL-17 (95, 96). Th 17 cells likely play a role in rosacea through two main mechanisms. They can increase neutrophil recruitment, helping to maintain inflammatory papules and pustules (97). In addition, they may drive VEGF expression, which supports dermal vascular growth and activation, ultimately contributing to angiogenesis and vascular dilation (98). In this context, the shared upstream mechanisms do not occur in an isolated inflammatory environment, but rather within a Th 1/Th 17-enriched microenvironment accompanied by excessive LL-37 production. Here, CGRP-driven vasodilation is amplified by LL-37-mediated VEGF upregulation via the mTORC1 and JAK2/STAT3 pathways, promoting sustained angiogenesis and structural vascular remodeling. This ultimately manifests as fixed telangiectasia and persistent centrofacial erythema that persists even in the absence of acute triggers.

### 3.4 Therapeutic implications of immune microenvironment divergence

This three-layer framework provides a rational basis treatment selection (As shown in **Table 2**). Interventions aimed at shared upstream triggers (e.g., microbiome modulation or TLR inhibition) may reduce the overall inflammatory load, but they are unlikely to have a disease-specific effect because these signals are common across multiple dermatoses. Targeting shared amplification modules [mast cell stabilization, TRP modulation, and VEGF inhibition] can alleviate overlapping vascular–inflammatory symptoms such as erythema and flushing. However, the magnitude and quality of the clinical response are determined by the dominant immune microenvironment, which shapes immune polarization and subsequently directs downstream vascular and pruritogenic programs. Consistently, Th2-targeted therapy (e.g., dupilumab) is highly effective in AD, but may disturb the local Th2/Th 1 balance and reveal rosacea-prone features. Conversely, agents targeting Th 1/Th 17 or LL-37 can reduce inflammatory and angiogenic drivers in rosacea-like settings, but do not directly address the barrier dysfunction and pruritogenic program that are central to AD. Overall, despite overlap in upstream triggers and amplification modules, AD and rosacea require fundamentally different, polarization-context-specific management strategies.

**Table 2:** Mechanistic basis for therapeutic differences between atopic dermatitis and rosacea.

*Therapeutic domain*	Atopic dermatitis	Rosacea	Critical note
*Dominant immune axis*	Th2/ILC2 → IL-4/IL-13/IL-31	Th1/Th17 + LL-37 innate axis •	Targeting one may unmask the other
*Barrier-targeted therapy*	Essential (emollients, filaggrin repair)	Hydration with Additional Cosmetic Actives and Functions Sunscreen (104)	AD barrier defect is disease-defining
*Th2 cytokine blockade*	Dupilumab Lebrikizumab Tralokinumab (105)	risk of paradoxical rosacea-like dermatitis	Dupilumab-induced rosacea-like dermatitis reported in up to ~5% of treated AD patients
*JAK inhibition dermatitis*	Upadacitinib, abrocitinib: targets IL-4/IL-13/IL-31 signaling; effective	Tofacitinib Upadacitinib Abrocitinib retrospective cohort and case reports (8)	Rosacea: JAK2/3−STAT dominant AD: JAK1−mediated Th2 cytokine signaling
*IL-31/itch pathway*	Nemolizumab (IL-31Rα): directly targets pruritus axis (105)	No established role	
*LL37-related signaling pathways*	not a primary target	TLR2,KLK5,LL-37,MMPs--Retinoids, Azelaicacid, Doxycycline, Carvedilol, Ivermectin mTORC1--Rapamycin, Celastrol (36) VEGF--Topical dobesilate, Tranexamic acid	LL-37 paradoxically antipruritic (semaphorin 3A) in atopic dermatitis
*Microbiome modulation*	Probiotics, S. epidermidis restoration Not a primary strategy	Demodex reduction; gut microbiome modulation Topical ivermectin (8) oral ivermectin/doxycycline	Disease-specific
*Th1/Th17-IL17*	Not a primary strategy	Secukinumab Aspirin Thalidomid (36)	
*TRP channel modulation*	•	capsazepine 4-t-butylcyclohexano (8)	Shared molecular target but different clinical endpoints

## 4 Conclusions and future perspectives

Atopic dermatitis (AD) and rosacea are common chronic inflammatory skin disorders traditionally regarded as distinct disease entities. However, accumulating epidemiological, clinical, and basic research evidence reveals significant overlap in both pathophysiological mechanisms and clinical manifestations between these conditions. Shared pathological features include microbiome alterations, aberrant immune responses, and neurovascular dysregulation. Clinically, the differential diagnosis between AD and rosacea remains particularly challenging in adult facial dermatitis, and their coexistence is not uncommon, further compounding disease burden. Currently, prospective cohort studies elucidating the true epidemiological characteristics and risk factors for AD-rosacea comorbidity are lacking. Nevertheless, the shared clinical trajectory-characterized by high prevalence, physiological-psychological vicious cycles, and substantial economic burden-mandates more comprehensive and standardized management approaches.

With advancements in specialized clinics and chronic disease management programs, future initiatives should focus on comorbidity risk assessment to enable precision management strategies that alleviate disease burden. Future research directions should prioritize (1):employing multi-omics technologies (genomics, transcriptomics, proteomics, microbiomics) to delineate shared and distinct pathogenic pathways (2); developing novel targeted therapeutics for common pathways (3); establishing optimized diagnostic criteria and evidence-based treatment guidelines grounded in pathophysiological features, utilizing advanced imaging modalities including dermatoscopy and video capillaroscopy (4); exploring personalized treatment strategies and predicting therapeutic responses based on individual molecular profiles.
